# Sex-Specific Dynamics of Global Chromatin Changes in Fetal Mouse Germ Cells

**DOI:** 10.1371/journal.pone.0023848

**Published:** 2011-08-19

**Authors:** Masanobu Abe, Shirley Y. Tsai, Seung-Gi Jin, Gerd P. Pfeifer, Piroska E. Szabó

**Affiliations:** 1 Department of Molecular and Cellular Biology, City of Hope National Medical Center and Beckman Research Institute, Duarte, California, United States of America; 2 Department of Cancer Biology, City of Hope National Medical Center and Beckman Research Institute, Duarte, California, United States of America; Bellvitge Biomedical Research Institute (IDIBELL), Spain

## Abstract

Mammalian germ cells undergo global reprogramming of DNA methylation during their development. Global DNA demethylation occurs around the time when the primordial germ cells colonize the embryonic gonads and this coincides with dynamic changes in chromatin composition. Global *de novo* DNA methylation takes place with remarkably different dynamics between the two sexes, prospermatogonia attaining methylation during fetal stages and oocytes attaining methylation postnatally. Our hypothesis was that dynamic changes in chromatin composition may precede or accompany the wave of global DNA *de novo* methylation as well. We used immunocytochemistry to measure global DNA methylation and chromatin components in male and female mouse fetal germ cells compared to control somatic cells of the gonad. We found that global DNA methylation levels sharply increased in male germ cells at 17.5 days post coitum, but remained low in female germ cells at all fetal stages. Global changes in chromatin composition: i, preceded global DNA methylation in fetal germ cells; ii, sex specifically occurred in male but not in female germ cells; iii, affected active and repressive histone marks and iv, included histone tail and histone globular domain modifications. Our data suggest that dynamic changes of chromatin composition may provide a framework for the pattern of male-specific de novo DNA methylation in prospermatogonia.

## Introduction

### Global waves of epigenetic changes during development

At two times during development, in the germ line and during preimplantation, the epigenome of mammals undergoes global resetting [1], [2], [3]. Global epigenetic reprogramming includes erasure of existing DNA methylation and establishment of new DNA methylation [4], [5]. Global demethylation occurs in primordial germ cells (PGCs) around the time when they colonize the genital ridges. Global *de novo* DNA methylation occurs with different dynamics between the two sexes. Prospermatogonia attain methylation during fetal stages and oocytes attain methylation postnatally. Immunostaining experiments revealed that dynamic and orderly chromatin changes take place in PGCs at the time of their specifications and during the time when DNA methylation erasure occurs [6], [7], [8], [9], [10]. Specifically, heterochromatin marks H3K9me2, H3K9me3 and H3K64me3 are transiently lost from PGCs at 8.0, 11.5 and 12.5 days post coitum (dpc), respectively [6], [7]. Little is known, however, about global chromatin changes during fetal germ cell development, and the information is limited to spatial reorganization of heterochromatin components H3K9me3 and CBX5 (HP1α) [10].

The information regarding global DNA methylation establishment in fetal germ cells mainly comes from bisulfite sequencing experiments at differentially methylated regions (DMRs) of imprinted genes and at repeat elements. Imprinted genes are expressed from one of the parental alleles in the soma [11], [12], whereas imprinted expression is neutralized in the germ line [13], [14]. Gametic imprints [15] —DNA methylation marks at DMRs that originate in sperm or oocyte— are critical for allele-specific monoallelic expression of imprinted genes in the soma [16], [17], [18], [19], [20]. Parental-specific methylation is maintained at DMRs in somatic cells during the life of the individual, but is reset (erased and reestablished) in the germ line according to the sex of the individual. In the mouse, DMRs become demethylated in primordial germ cells (PGCs), between 9.5 and 12.5 dpc [4], [21]. Remethylation of paternal DMRs begins at 14.5 dpc in prospermatogonia [22], [23], [24], [25], and of maternal DMRs after birth in growing oocytes [26], [27]. The mechanism of imprint establishment is not fully understood, but non-coding RNA targeting by piRNA [28], tandem repeats [29] that serve as promoter for non-coding RNA [28], transcription-related events [30], and a chromatin modifying protein [31] play role in the process.

DNA and histone methylation are structurally and functionally linked [32]. Mammalian DNA methylation depends on the methylation status of H3K4 and H3K9 residues. H3K9 methylation by SUV39H1 is required for DNA methylation at the pericentric heterochromatin in mice [33]. Significantly, the H3K4 demethylase KDM1B is required for the establishment of maternal methylation imprints at some DMRs in oocytes [31]. The DNA *de novo* methylation cofactor, DNMT3L [34] requires a DNA substrate associated with unmethylated H3K4 [35]. It is not known if the presence of any histone mark is required for global *de novo* methylation or for imprint establishment. We hypothesized that chromatin composition may provide a framework for global de novo methylation and may trigger imprint establishment in the germ line. We used immunocytochemistry to examine global changes of DNA methylation and chromatin composition in male and female mouse fetal germ cells and somatic cells of the gonad. We expected that fetal germ cells exhibit dynamic changes in chromatin composition compared to somatic cells of the gonad because the latter are not known to undergo global epigenetic remodeling. We also expected that the global chromatin changes will be sex-specific: they will be more pronounced in male than female fetal germ cells, because at fetal stages only the male but not the female germ line undergoes global *de novo* DNA methylation.

## Results

### DNA methylation

**Figure 1:**
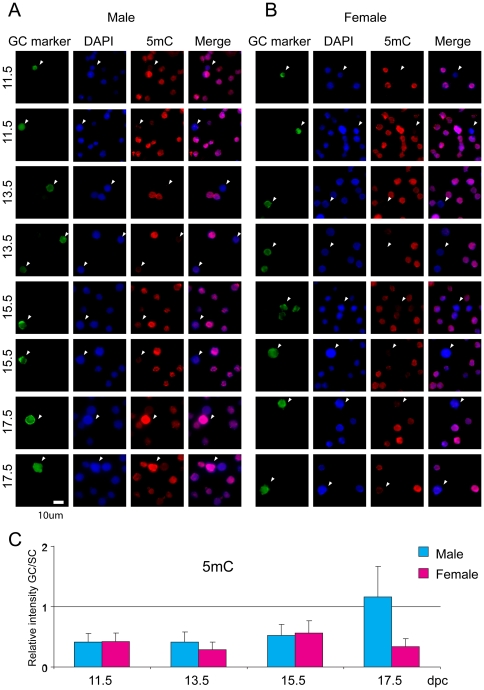
Global changes of DNA methylation in fetal germ cells. Germ cells (marked by arrowheads) distinctly stain positive using germ cell markers, Oct3/Oct4 or Ddx4. The Oct3/Oct4 marker was used at 11.5 dpc and the Ddx4 marker was used at later stages. Anti-5mC antibody was used to stain methylated DNA. Anti-DNA antibody was used to visualize nuclei. Cells were obtained from (A) Male gonads (B) Female gonads. Duplicates are shown for each developmental stage, indicated to the left in dpc. (C) The relative staining intensities of female and male germ cells (GC) to the respective gonadal somatic cells (SC) are depicted in the chart with standard deviations.

We visualized DNA methylation by staining germ cells and somatic cells of the developing male and female fetal mouse gonad at different developmental stages using an anti-5-methyl cytosine (5mC) antibody (Figure 1). Germ cells were distinguished by double staining with antibodies against germ cell markers Oct3/Oct4 or Ddx4. We found that 5mC levels were very low compared to somatic cells in both male and female germ cells between 11.5 and 15.5 dpc. The global 5mC level sharply increased in male germ cells at 17.5 dpc but remained low in female germ cells at all stages. These results were in agreement with previous observations of methylation erasure and establishment using bisulfite sequencing [4], [21], [22], [23], [24], [36].

### Active histone tail marks

**Figure 2 pone-0023848-g002:**
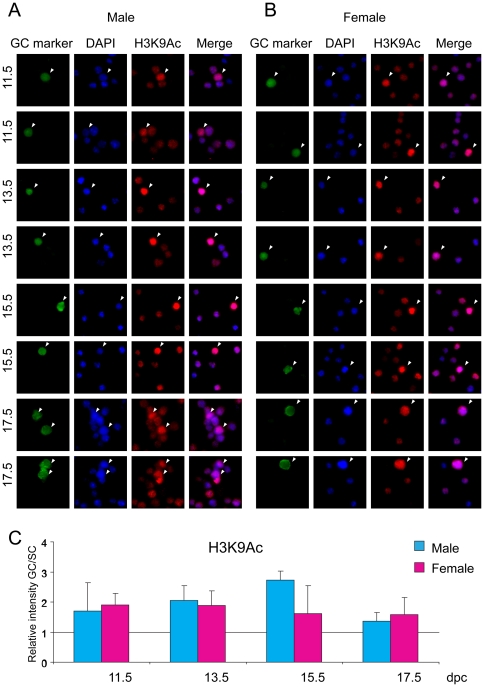
Global changes of H3K9ac in fetal germ cells. Gonad preparations were stained with anti-H3K9ac antibody. DAPI staining was used to visualize nuclei (blue). Other details are as in legend to Figure 1.

**Figure 3 pone-0023848-g003:**
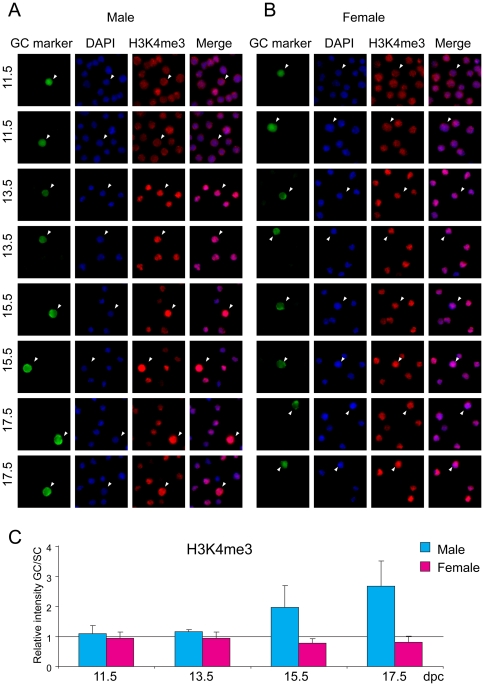
Global changes of H3K4me3 in fetal germ cells. Gonad preparations were stained with anti-H3K4me3 antibody. DAPI was used to visualize nuclei (blue). Other details are as in legend to Figure 1.

Acetylation of residue K9 and trimethylation of residue K4 in the tail of histone H3 are well-known components of active chromatin. Germ cells and somatic cells of the fetal gonad were stained with antibodies against H3K9ac and H3K4me3. Male and female germ cells stained strongly for H3K9ac compared to somatic cells at each developmental stage (Figure 2). Male germ cells reached the highest level of H3K9ac at 15.5 dpc. No peak was observed in female germ cells. H3K4me3 levels were very similar between germ cells and somatic cells in the female throughout fetal development and in the male at 11.5–13.5 dpc (Figure 3). H3K4me3 levels increased in male germ cells at 15.5 and reached even higher levels at 17.5 dpc.

### Repressive histone tail marks

**Figure 4 pone-0023848-g004:**
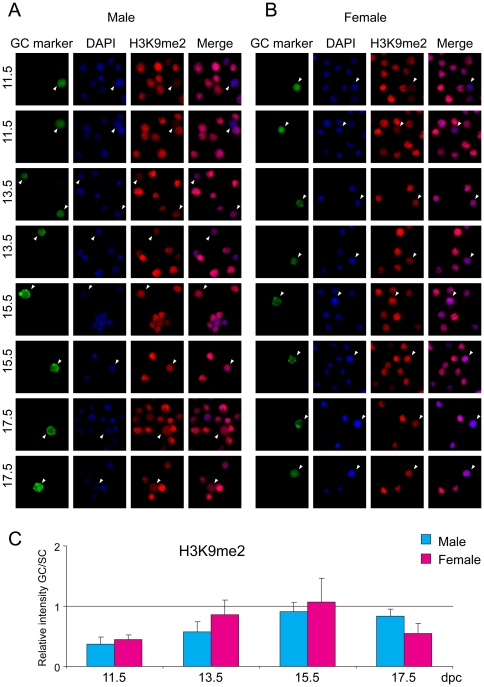
Global changes of H3K9me2 in fetal germ cells. Gonad preparations were stained with anti-H3K9me2 antibody. DAPI was used to visualize nuclei (blue). Other details are as in legend to Figure 1.

**Figure 5 pone-0023848-g005:**
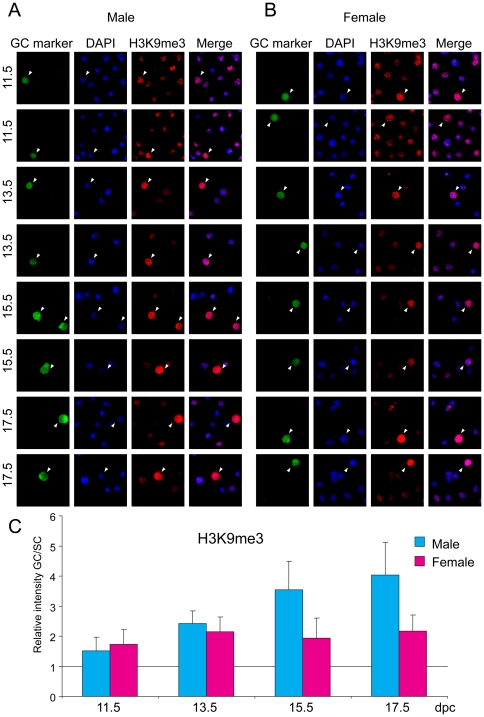
Global changes of H3K9me3 in fetal germ cells. Gonad preparations were stained with anti-H3K9me3 antibody. DAPI was used to visualize nuclei (blue). Other details are as in legend to Figure 1.

**Figure 6 pone-0023848-g006:**
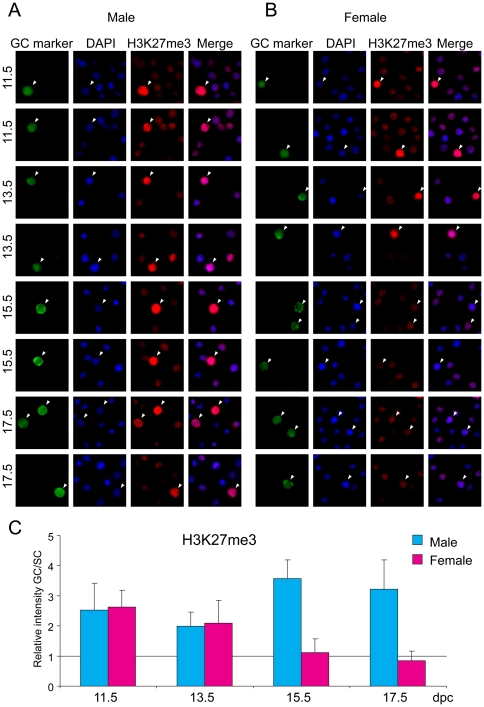
Global changes of H3K27me3 in fetal germ cells. Gonad preparations were stained with anti-H3K27me3 antibody. DAPI was used to visualize nuclei (blue). Other details are as in legend to Figure 1.

Di-and trimethylation of residue K9 and trimethylation of residue K27 in the tail of histone H3 are well-known components of repressive chromatin. Germ cells and somatic cells of the fetal gonad were stained with antibodies against chromatin components H3K9me2, H3K9me3 and H3K27me3. Fetal germ cells stained weakly for H3K9me2 compared to somatic cells at each developmental stage (Figure 4) except at 15.5 dpc, when both male and female germ cells reached the staining level of somatic cells. H3K9me3 levels were higher in gem cells of both sexes than in somatic cells throughout fetal development (Figure 5). H3K9me3 levels increased in male germ cells at 15.5 and 17.5 dpc. H3K27me3 was high in male and female germ cells compared to the respective somatic cells at 11.5 and 13.5 dpc (Figure 6), when DNA methylation is the lowest. This is in agreement with previous reports that showed increased H3K27me3 levels in PGCs between 9.5 and 11.5 dpc [8], [9]. It is tempting to speculate that Polycomb-mediated repression may be the major repressive force in midgestation germ cells at the time when DNA methylation is globally erased. At 15.5 dpc there was a dramatic increase in H3K27me3 levels in male germ cells, but at the same time H3K27me3 levels dropped in female germ cells.

### Histone globular domain marks in fetal germ cells

**Figure 7 pone-0023848-g007:**
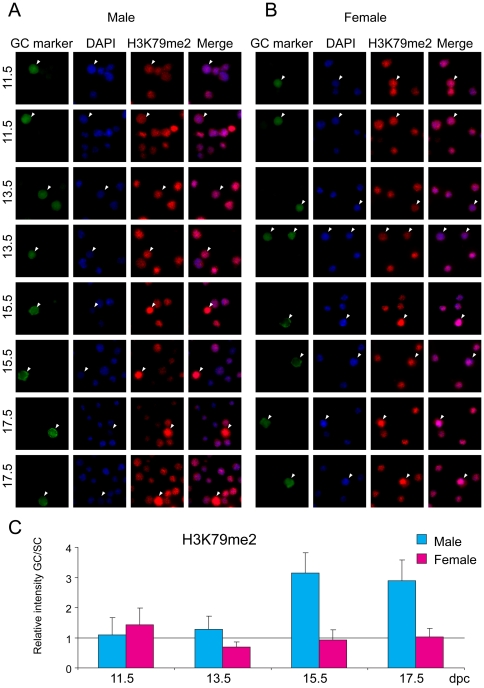
Global changes of H3K79me2 in fetal germ cells. Gonad preparations were stained with anti-H3K79me2 antibody. DAPI was used to visualize nuclei (blue). Other details are as in legend to Figure 1.

**Figure 8:**
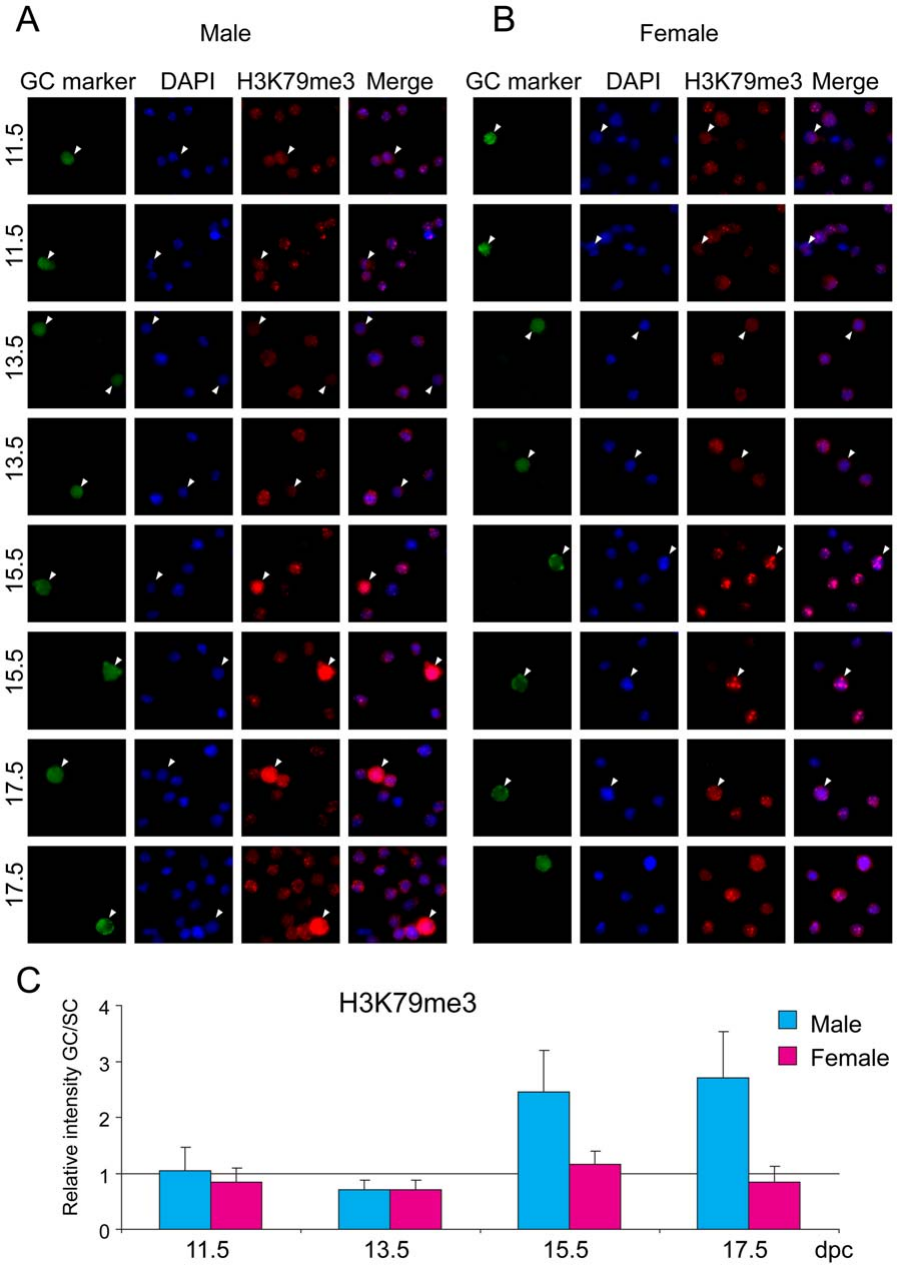
Global changes of H3K79me3 in fetal germ cells. Gonad preparations were stained with anti-H3K79me3 antibody. DAPI was used to visualize nuclei (blue). Other details are as in legend to Figure 1.

**Figure 9:**
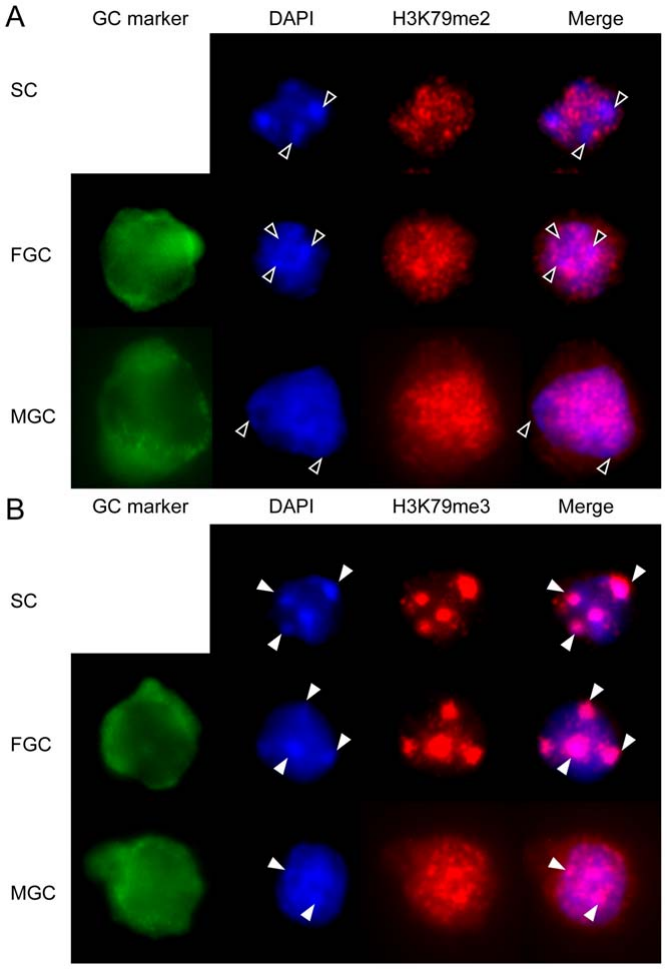
Nuclear distribution of H3K79me2 and H3K79me3 in fetal germ cells. Female and male germ cells (FGC and MGC, respectively) and female somatic cells (SC) at 15.5 dpc were stained with the H3K79me2 and H3K79me3 antibodies as indicated and are shown in the different channels separately and in red-blue composite. (A) DAPI-stained chromocenters are excluded from H3K79me2 signals (open arrowheads). (B) DAPI-rich chromocenters colocalize with H3K79me3 signals (closed arrowheads). Other details are as in legend to Figure 1.

Di-and trimethylation of residue K79 in the globular domain of histone H3 are interesting chromatin components, with somewhat debated and perhaps diverse functions between species [37]. H3K79me2 and H3K79me3 are associated with the DNA-unmethylated and methylated alleles of DMRs, respectively [38]. H3K79me2 and also H3K79me3 levels were very similar between germ cells and somatic cells in the female throughout fetal development and in the male at 11.5–13.5 dpc (Figure 7 and 8). Levels of both H3K79me2 and H3K79me3 sharply increased in male germ cells at 15.5 and remained high at 17.5 dpc. These two marks, however, showed complementary nuclear staining patterns (Figure 9) similarly to what was found in fibroblasts and oocytes [39]. Female germ cells and somatic cells have several large DAPI-positive compartments (Figure 9) and H3K79me2 excluded these DAPI-rich regions whereas H3K79me3 co-localized with DAPI signals. Male fetal germ cells have less clear chromocenters [10], but the weak DAPI spots largely colocalized with H3K79me3 and did not colocalize with H3K79me2 (Figure 9).

## Discussion

### Dynamic global chromatin changes in fetal germ cells

**Figure 10:**
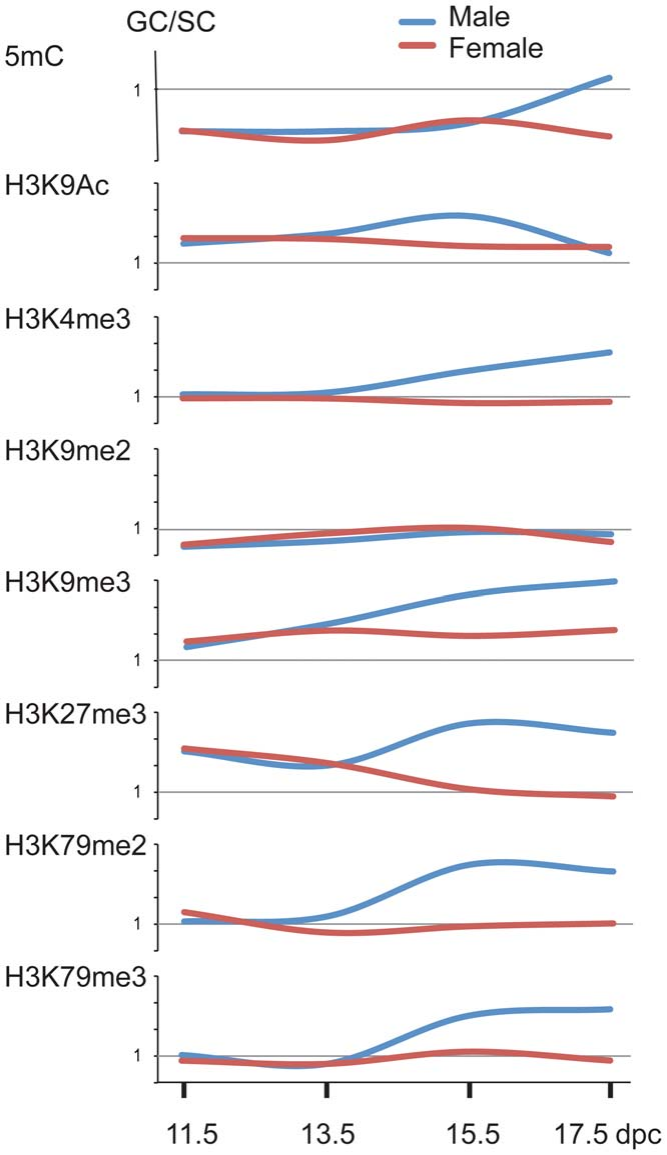
Summary of global epigenetic changes in fetal germ cells. The relative staining intensities of germ cells versus somatic germ cells (GC/SC) were combined from Figures 1–8. The data points at different fetal stages (in dpc) were connected to show the general trends in global chromatin changes. Male fetal germ cells (blue lines) exhibit strong increase in both active and repressive histone marks at 15.5–17.5 dpc, preceding the peak of global de novo DNA methylation that occurs at 17.5 dpc. Female germ cells (red lines) do not undergo global de novo methylation and do not exhibit global chromatin changes during fetal development.

The major finding of this study is that chromatin undergoes dynamic global changes in male fetal germ cells at the time of global DNA methylation reprogramming (Figure 10). The dramatic chromatin changes in prospermatogonia occurred at 15.5 dpc and thus preceded the wave of CpG methylation peak at 17.5 dpc. Importantly, global chromatin changes were not apparent in female fetal germ cells that do not accumulate DNA methylation until later, at the growing oocyte stage. The global increase in prospermatogonia was not limited to heterochromatin components, but also included marks that are typically found in euchromatin. Particularly interesting is that global levels of globular histone marks H3K79me3 and H3K79me2 strongly increased at 15.5 dpc. H3K79me3 and H3K79me2 occupy reciprocal, methylated versus unmethylated parental alleles at DMRs [38] and generally cluster with repressive and active histone marks, respectively in chromosome-wide mapping [40]. They also globally occupy different nuclear regions, H3K79me3 is localized to DAPI-stained heterochromatin whereas H3K79me2 is localized to DAPI-poor regions (Figure 9) and [39]. It is possible that newly formed chromatin patterns (for example H3K79me3-H3K9me3 versus H3K79me2-H3K4me3-rich regions) target and repel *de novo* DNA methylation at specific genomic regions, respectively and thus help establish the prospermatogonia-specific DNA methylation pattern. Our recent study provided the first information on chromatin at a specific locus in gestational age germ cells. We showed that at the *H19/Igf2* gametic DMR, the erasure of allele-specific chromatin follows, and therefore, is not required for CpG demethylation in PGCs [24]. No data exists, however, on chromatin at single loci during the DNA remethylation stage. It will be important to perform chromatin mapping in fetal germ cells at specific loci and at a genome-wide scale to precisely reveal the patterning effect of chromatin on *de novo* DNA methylation.

Similarly to fetal male germ cells, dynamic chromatin changes may also occur in the growing oocytes of the adult mouse before and during the establishment of de novo methylation in the female germ line.

## Materials and Methods

### Ethics statement

The experiments involving mice had been approved by the IACUC of the City of Hope under protocol ID 91023. Housing and care of the animals has been consistent with the Public Health Service Policy, the NIH “Guide for the Care and Use of Laboratory Animals” and the Animal Welfare Act.

### Embryo isolation and staging

Female CF1 (Charles River Laboratories) mice were mated with male OG2 [13] mice. Genital ridges were dissected at 11.5 dpc. Gonads were dissected from male and female fetuses at 13.5, 15.5 and 17.5 dpc. Male and female gonads were distinguished by their distinct morphology at 13.5, 15.5 and 17.5 dpc. At 11.5 dpc the sex of the embryo was detemined by PCR. Gonads were incubated at 37 C for 15 m in Trypsin-EDTA and triturated to achieve a single cell suspension containing germ cells and somatic cells. Dulbecco's Modified Eagle's Medium (DMEM) (Invitrogen, Carlsbad, CA) supplemented with 20% FBS was added to inactivate trypsin.

### Sex-specific PCR

At 11.5 dpc sex of the embryo was determined by PCR amplification of two genes in a single reaction. The *Sry* amplicon indicated the presence of Y chromosome and male sex, whereas amplification of the *Snrpn* gene served as a positive control. Primer sequences were as follows: *Sry* forward: 5′-ATGGAGGGCCATGTCAAGCG-3′; *Sry* reverse: 5′-TGCCACTCCTCTGTGACACTTTAG-3′; *Snrpn* forward: 5′-CTTTTGGTAGCTGCCTTTTGG-3′ and *Snrpn* reverse: 5′-CTAGTCTTGCCGCAATGGCTC-3′. Embryo tails were boiled at 95°C for 10 min in distilled water and the genomic DNA was used for template in PCR. The PCR condition was as follows; 95°C for 2 min, 95°C for 30 s, 58°C for 45 s, 72°C for 40 s (31 cycles), followed by 72°C for 5 min.

### Antibodies

The following antibodies were used for immunostaining (dilution and source indicated in parentheses): rabbit anti-H3K9me2 (1/1000, Upstate 07-441); rabbit anti-H3K9me3 (1/1000, Upstate 17-625); rabbit anti-H3K9ac (1/2000, Upstate 07-352); rabbit anti-H3K4me3 (1/2000, Upstate 07-473); rabbit anti-H3K27me3 (1/2000, Upstate 07-449); rat anti-Oct3/4 (1/1000, RD systems MAB1759); mouse anti-5-MeC (1/500, Eurogentec 900 P-A); mouse anti-DNA (1/200, QED 12404); rabbit anti-Ddx4/Mvh (1/2000, Abcam ab13840-100); rabbit anti-H3K79me2 (1/2000, Upstate 07-366) and rabbit anti-H3K79me3 (1/2000, Abcam ab2621). The following secondary antibodies from Molecular Probes were used at a 1/1000 dilution: Alexa Fluor 488 goat anti-rat IgG; Alexa Fluor 488 goat anti-rabbit IgG; Alexa Fluor 568 goat anti-rabbit IgG; Alexa Fluor 568 goat anti-mouse IgG; Alexa Fluor 350 goat anti-mouse IgG.

### Immunofluorescence staining of chromatin marks

The genital ridges or gonads of the relevant developmental stages were trypsinised and the single cells suspension was settled on the poly-L-lysine (Sigma Aldrich, St. Louis, MO) treated slides in DMEM supplemented with 10% FBS in a CO_2_ incubator. The cells were briefly washed with 1xPBS and fixed in 4% PFA in PBS for 15 min at room temperature. (I) The cells were permeabilised for 10 min using PBS, 0.2% Triton X-100 and were blocked for 1 hr in PBST (PBS, 0.05% Tween20), 1% BSA at room temperature. (II) The antibody staining was carried out for 1 hr in PBST. (III) The slides were subsequently washed 3× in PBST (10 min each wash) and (IV) incubated with Alexa dye-conjugated secondary antibodies (Molecular Probes) for 1 hr at room temperature in the dark, and (VI) washed 5×5 min in PBST. The slides were subsequently washed for 3×10 min in PBST. (VII) Cells were blocked in PBST, 1% BSA at 4°C overnight, followed by the steps II to VI for staining with germ cell specific antibodies (Oct-3/4 or Ddx4). The slides were then mounted in ProLong Gold Antifade Reagent with DAPI (Invitrogen) and imaged using IX81 inverted microscope (Olympus). During and after applying Alexa dyes conjugated secondary antibodies, specimens were kept in the dark.

### 5mC staining

After performing the above steps I to VI for germ cell-specific antibodies (Oct-3/4 or Ddx4), the cells were permeabilized for 30 min using PBS, 0.2% Triton X-100 and were treated with RNase A (100 µg/ml) for 30 min at room temperature and washed 2×5 min in PBS. DNA was denatured 10 min 4 N HCl, 0.1% Triton X-100 and washed 2×5 min in PBS. The cells were neutralized for 30 min in 100 mM Tris HCl, pH 8.0 and washed 2×5 min in PBS. The cells were blocked in PBST, 1% BSA at 4°C overnight. The steps from II to VI were performed for 5mC antibody, and blocked for 1 hr in PBST, 1% BSA at room temperature. The anti-DNA antibody staining was carried out in PBST at 4°C overnight, followed by the steps from II to VI. The slides were then mounted in ProLong Gold Antifade Reagent (Invitrogen).

### Quantitation

The quantification of staining intensity was performed using Image-Pro Plus version 6.3 software (Media Cybernetics, Carlsbad, CA). Mean fluorescence intensity/area values were measured for 20–30 germ cells (green) and 200–300 somatic cells. After background correction, red fluorescence values were divided by DAPI intensity values for each cell. The average germ cell red/blue value was then divided by the somatic cell average red/DAPI value to obtain the relative value of GC/SC. Standard deviation values were calculated from the individual GC values over the average SC values [8]. The results were in agreement between two independent sets of experiments that included gonad collection, staining, microscopy and quantification, done by two investigators (Figure S1).

## Supporting Information

Figure S1 **Reproducubility of the image quantitation method.** Image quantification results obtained in two independent experiments by two investigators (A) MA and (B) ST are shown for two antibodies, H3K79me2 and H3K79me3, at 17.5 dpc. The experiments included fetal gonad collection, immunostaining, microscopy and quantification. Mean fluorescence intensity/area values were measured for 20–30 male or female germ cells (MGC and FGC) and 200–300 male or female somatic cells (MSC, FSC). After background correction, red fluorescence values were divided by DAPI intensity values for germ cells and somatic cells separately. Standard deviation values were calculated from the individual GC values over the average SC values. Male germ cells exhibited statistically significant difference from somatic cells in global H3K79me2 and H3K79me3 staining according to T-tests (***p-value<0.000001). (C) The average germ cell (GC) red/blue value was then divided by the somatic cell (SC) average red/DAPI value to obtain the relative value of GC/SC for each experiment. The ratios obtained in measurement A and B were similar.
(PDF)
