# Supplementary figures and images for: Sex-Specific Dynamics of Global Chromatin Changes in Fetal Mouse Germ Cells
Source: PLoS One. 2011 Aug 19;6(8):e23848. doi: 10.1371/journal.pone.0023848 (PMC3158789; doi:10.1371/journal.pone.0023848)

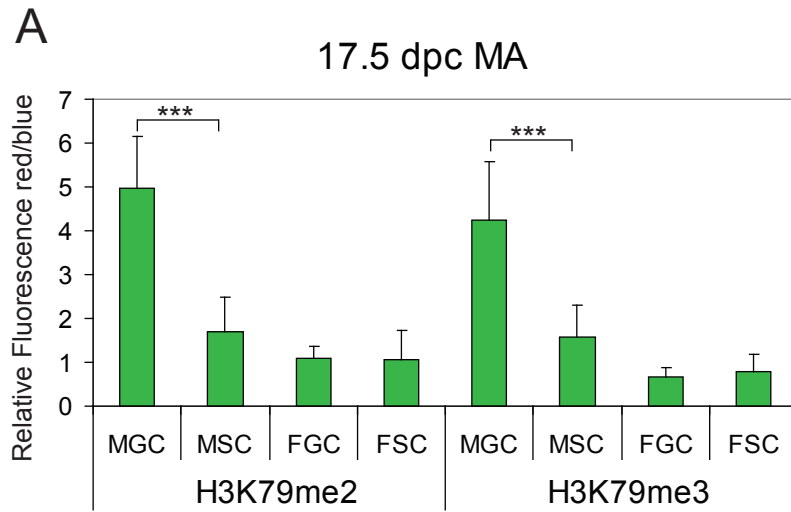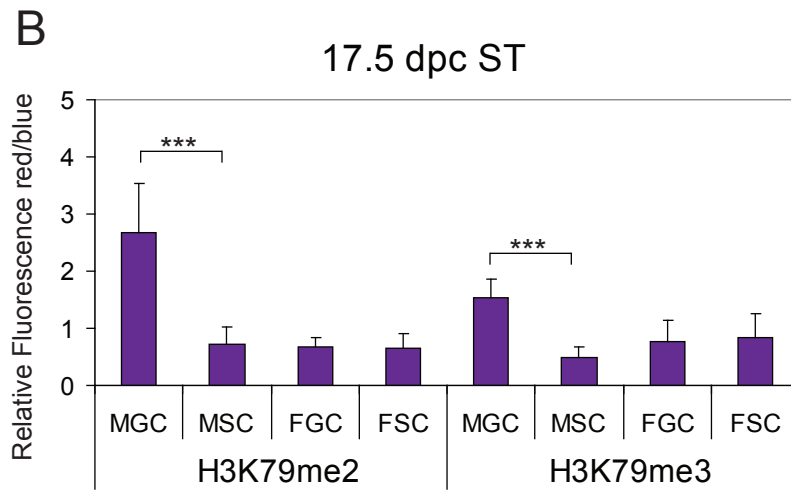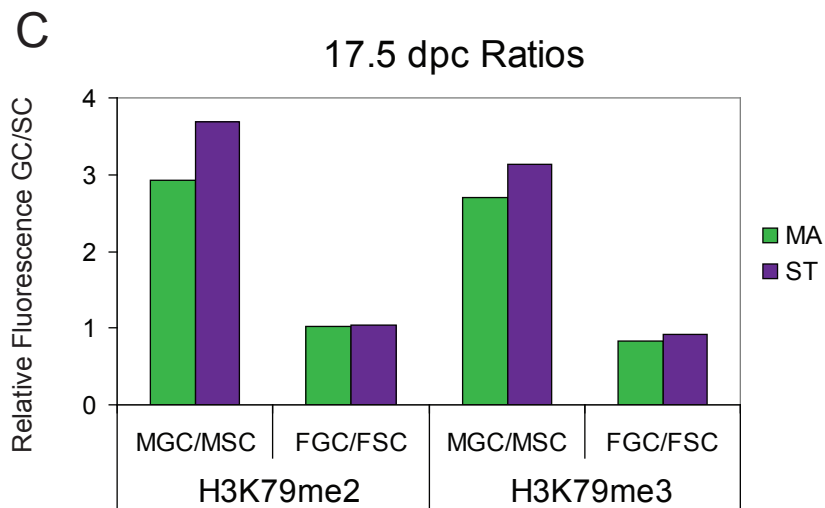

Supplement: Figure S1 — Reproducubility of the image quantitation method. Image quantification results obtained in two independent experiments by two investigators (A) MA and (B) ST are shown for two antibodies, H3K79me2 and H3K79me3, at 17.5 dpc. The experiments included fetal gonad collection, immunostaining, microscopy and quantification. Mean fluorescence intensity/area values were measured for 20–30 male or female germ cells (MGC and FGC) and 200–300 male or female somatic cells (MSC, FSC). After background correction, red fluorescence values were divided by DAPI intensity values for germ cells and somatic cells separately. Standard deviation values were calculated from the individual GC values over the average SC values. Male germ cells exhibited statistically significant difference from somatic cells in global H3K79me2 and H3K79me3 staining according to T-tests (***p-value<0.000001). (C) The average germ cell (GC) red/blue value was then divided by the somatic cell (SC) average red/DAPI value to obtain the relative value of GC/SC for each experiment. The ratios obtained in measurement A and B were similar. (PDF) [file pone.0023848.s001.pdf]
